# Painless Takotsubo Syndrome Triggered by Acute Exacerbation of Chronic Obstructive Pulmonary Disease (COPD): A Retrospective Observational Study of Seven Cases

**DOI:** 10.7759/cureus.119535

**Published:** 2026-11-12

**Authors:** Ciro Kirchenchtejn, Alan Besborodco, Marcelo Wolff Fridman, Luisa H Millner

**Affiliations:** 1 Medicine, Universidade Federal de São Paulo (Unifesp), São Paulo, BRA; 2 Internal Medicine, Universidade Nove de Julho, São Paulo, BRA; 3 Gastroenterology, Universidade de Caxias do Sul, Porto Alegre, BRA; 4 Internal Medicine, Hospital Israelita Albert Einstein, São Paulo, BRA

**Keywords:** catecholamine excess, chronic obstructive pulmonary disease (copd), emergency medicine management, heart failure, takotsubo cardiomyopathy (tc)

## Abstract

Takotsubo syndrome (TS) as a manifestation of acute chronic obstructive pulmonary disease (COPD) exacerbation is uncommonly reported. Emotional and adrenergic stress, hypoxemia, negative intrathoracic pressure, and the use of beta-agonists are frequently implicated in triggering myocardial dysfunction in this setting. We present a retrospective study of seven cases of TS in patients with COPD exacerbation in which none reported typical chest pain, representing a diagnostic challenge in patients with an apparent clinical justification for their dyspnea. All patients had electrocardiographic changes and elevated cardiac biomarkers, underwent echocardiography and coronary angiography or coronary computed tomography angiography, and received a confirmed diagnosis of TS. Five patients achieved complete recovery of ventricular function, one had partial recovery, and one died during hospitalization due to refractory ventricular arrhythmia. Systematic use of electrocardiography and cardiac biomarkers in COPD exacerbation enabled early diagnosis and appropriate management in the absence of typical angina.

## Introduction

The American Heart Association defines Takotsubo syndrome (TS) as an acute, potentially life-threatening acquired cardiomyopathy with a clinical presentation resembling myocardial infarction, whose natural history, treatment, and prognosis are not yet fully understood [1]. It accounts for approximately 1-2% of acute coronary syndrome presentations and is frequently associated with physical or emotional stressors as triggering factors [1].

A substantial proportion of secondary TS cases occur in patients already hospitalized for another medical, surgical, anesthetic, obstetric, or psychiatric condition. In these patients, sudden activation of the sympathetic nervous system or an increase in catecholamine levels precipitates TS as a complication of the underlying condition or its treatment [2]. Secondary TS affects men and women more equally than the primary form; is triggered by comorbid conditions such as pheochromocytoma, cocaine use, postoperative states, and asthma or chronic obstructive pulmonary disease (COPD) crises; and is associated with higher cardiac enzyme release (specifically high-sensitivity troponin and creatine kinase-MB (CK-MB)) and more severe or prolonged cardiac dysfunction [2].

The association between COPD and TS is established in the literature. Multiple studies have confirmed this relationship and point to sympathetic hyperactivity as the central mechanism, although gaps in the pathophysiology of this association persist [3,4,5]. In a Polish cohort following patients after TS compared to controls with acute coronary syndrome, a significantly higher prevalence of COPD was observed among TS patients (11.6% vs 1.0%; p = 0.002) [6]. Data from the National Inpatient Sample indicate that 21.8% of patients with TS had COPD, reinforcing its role as a relevant comorbidity. Patients hospitalized with TS and concurrent COPD show higher rates of in-hospital mortality, acute respiratory failure, cardiogenic shock, longer length of stay, and higher hospital costs compared to those without COPD [7]. Furthermore, a recent Heart & Lung study explicitly recognized COPD exacerbation as a physical trigger for secondary TS and demonstrated that patients presenting primarily with COPD exacerbation who develop TS have higher rates of respiratory failure and longer hospitalizations, though lower rates of cardiogenic shock, compared to patients presenting primarily for TS who have COPD as a comorbidity [4].

TS can be benign or potentially fatal depending on the triggering factor. A prognostic classification based on triggering factors has been proposed and may serve as a clinical tool to predict short- and long-term outcomes [8]. Specifically, patients with COPD and TS have a hazard ratio for mortality of 2.1 compared to controls without coronary artery disease, similar to the excess mortality observed in patients with established coronary artery disease [9]. The management of TS remains primarily supportive and is guided by observational data, registry analyses, and expert consensus rather than robust randomized controlled trials [10]. Beta-blocker therapy in TS patients has been associated with lower follow-up mortality, but not with lower TS recurrence [11]. The syndrome is susceptible to recurrence, and COPD patients may be at particular risk [5,12].

In Brazil, a case of TS associated with COPD exacerbation with typical angina presentation and evolution to cardiogenic shock was described, with administration of bronchodilators suggested as a possible precipitating factor [13].

Therefore, this study aims to evaluate the special clinical features, in-hospital evolution, and outcomes of a cohort of patients diagnosed with TS secondary to acute COPD exacerbation. In this context, we present seven cases of TS in patients who did not report chest pain at initial presentation, manifesting predominantly with symptoms of COPD exacerbation, and discuss the diagnostic approach, management, complications, and outcomes.

## Materials and methods

Study design and setting

This retrospective observational study evaluated medical records of patients admitted with TS triggered by an acute exacerbation of COPD between 2016 and 2026. Data were retrieved from the emergency department and inpatient records of Hospital Alemão Oswaldo Cruz, a tertiary private hospital located in São Paulo, Brazil. All cases were systematically reviewed and confirmed by the institution's cardiology department.

Eligibility criteria

Patients were eligible for inclusion if they were hospitalized for an acute exacerbation of COPD, defined according to the Global Initiative for Chronic Obstructive Lung Disease (GOLD) criteria [13], and if they met the International Takotsubo Diagnostic Criteria (InterTAK criteria) for TS [14]. The diagnosis of TS required transient left ventricular dysfunction (hypokinesia, akinesia, or dyskinesia) extending beyond a single epicardial coronary vascular territory, absence of culprit obstructive coronary artery disease on invasive coronary angiography or coronary computed tomography angiography (CCTA), new electrocardiographic abnormalities and/or dynamic elevation of cardiac biomarkers, and exclusion of myocarditis. All diagnoses of TS were formally confirmed by the attending cardiology team.

Patients were excluded when clinical, laboratory, or imaging data were insufficient to adequately characterize the case or when an alternative diagnosis, such as acute myocarditis or obstructive acute myocardial infarction, better explained the acute myocardial dysfunction.

Data collection and variables

Demographic characteristics, vital signs at presentation, and acute respiratory symptoms were recorded upon admission. A detailed pharmacological history was also collected, with particular attention to the use of short- and long-acting beta-agonists before and during hospitalization.

Diagnostic and clinical variables included electrocardiographic findings and cardiac biomarker measurements, including admission and peak levels of high-sensitivity troponin, CK-MB, and B-type natriuretic peptide (BNP). Imaging data included transthoracic echocardiographic parameters, such as left ventricular ejection fraction and regional wall motion abnormalities, as well as findings from invasive coronary angiography or CCTA and chest imaging, including radiography or high-resolution computed tomography. Clinical outcomes included intensive care unit (ICU) admission, need for mechanical ventilation, in-hospital complications, total length of hospital stay, documentation of myocardial recovery, and in-hospital mortality.

Myocardial recovery was systematically assessed on follow-up transthoracic echocardiography. Complete recovery was defined as normalization of left ventricular ejection fraction (LVEF ≥ 50%) with complete resolution of regional wall motion abnormalities. Partial recovery was defined as clinical and echocardiographic improvement (increased LVEF and reduced akinetic area) with persistent localized wall motion abnormalities.

Statistical analysis

Continuous variables were expressed as medians with interquartile ranges (IQR) or means with standard deviations, according to data distribution. Categorical variables were presented as absolute counts and percentages. Due to the sample size of this retrospective cohort, data analysis was strictly descriptive. Statistical calculations were performed using IBM SPSS Statistics version 28.0 (IBM Corp., Armonk, NY, USA).

Ethical considerations

This study was conducted in accordance with the ethical principles of the Declaration of Helsinki and local regulatory guidelines for clinical research. The study protocol was reviewed and approved by the Institutional Review Board (IRB) of Hospital Alemão Oswaldo Cruz under approval number 8.438.110. Written informed consent was obtained from active patients or legal representatives when feasible. For patients who could not be contacted due to loss to follow-up, the IRB granted a waiver of informed consent to allow anonymous medical record review.

## Results

A total of seven patients with a prior diagnosis of COPD were analyzed. Six patients presented with an FEV1 below 50% of the predicted value, characterizing severe to very severe airflow obstruction, whereas Case 6 presented with moderate airflow obstruction. The diagnosis of COPD in Case 6 was firmly established based on a consistent clinical history, a documented pulmonologist report confirming the condition, non-responsive spirometric values to bronchodilators, and high-resolution chest computed tomography demonstrating extensive emphysema and bronchial disease. Despite the difference in baseline spirometric severity (moderate versus severe/very severe), all patients, including Case 6, were classified as Group E (exacerbator) in accordance with the current GOLD guidelines, due to their history of exacerbations.

The mean age was 74.1 ± 6.0 years, with a female predominance of 85.7% (n = 6). All patients had a smoking history; however, only one patient (Case 3) was an active smoker at admission. The primary clinical presentation was worsening dyspnea and cough without typical angina in the majority of cases; only two patients (Cases 3 and 6) reported atypical chest discomfort without frank chest pain. All patients received high-dose inhaled and systemic bronchodilators along with corticosteroids prior to or during initial evaluation. During hospitalization, electrocardiographic changes combined with elevation of troponin or CK-MB levels raised the clinical suspicion of acute coronary syndrome, prompting serial cardiac biomarker measurements, echocardiograms, and coronary angiography or CCTA for diagnostic clarification. Segmental left ventricular dysfunction was observed on echocardiography in all patients. Coronary evaluation revealed no acute obstructive lesions compatible with the wall motion abnormalities, confirming the diagnosis of TS in all cases.

Representative diagnostic findings from Case 7 illustrate these characteristic features: a transthoracic echocardiogram demonstrating classic apical ballooning and akinesia typical of TS (Figure 1), followed by CCTA confirming the absence of acute obstructive coronary lesions (Figure 2).

**Figure 1:**
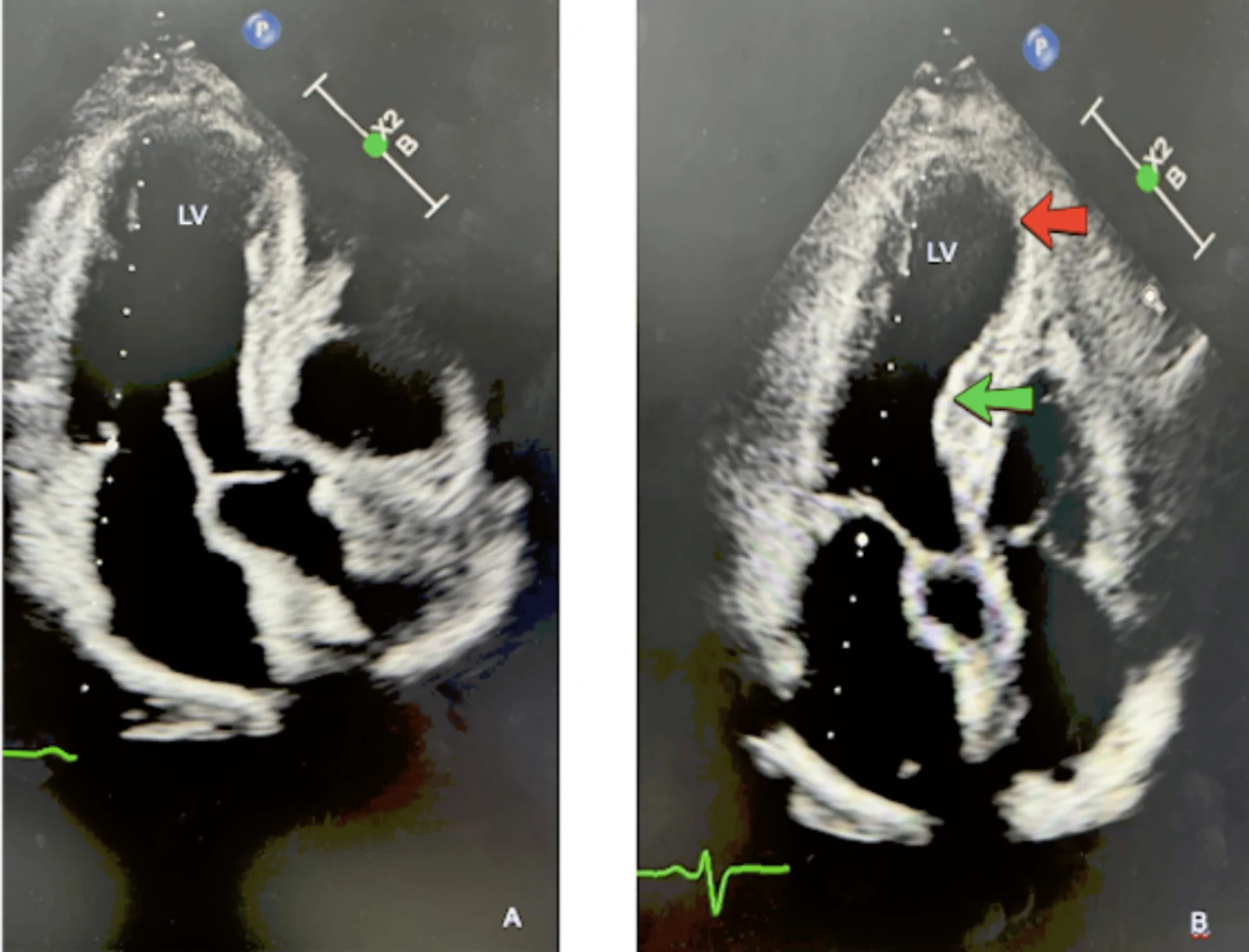
Transthoracic echocardiographic findings in Case 7. A woman with Takotsubo syndrome during an acute COPD exacerbation, presented without chest pain but with elevated cardiac biomarkers. (A) Left ventricle during diastole. (B) Left ventricle during systole with a kinetic and ballooned LV apex (red arrow) and hyperkinesis of the proximal ventricle (green arrow). LV: left ventricle; COPD: chronic obstructive pulmonary disease

**Figure 2:**
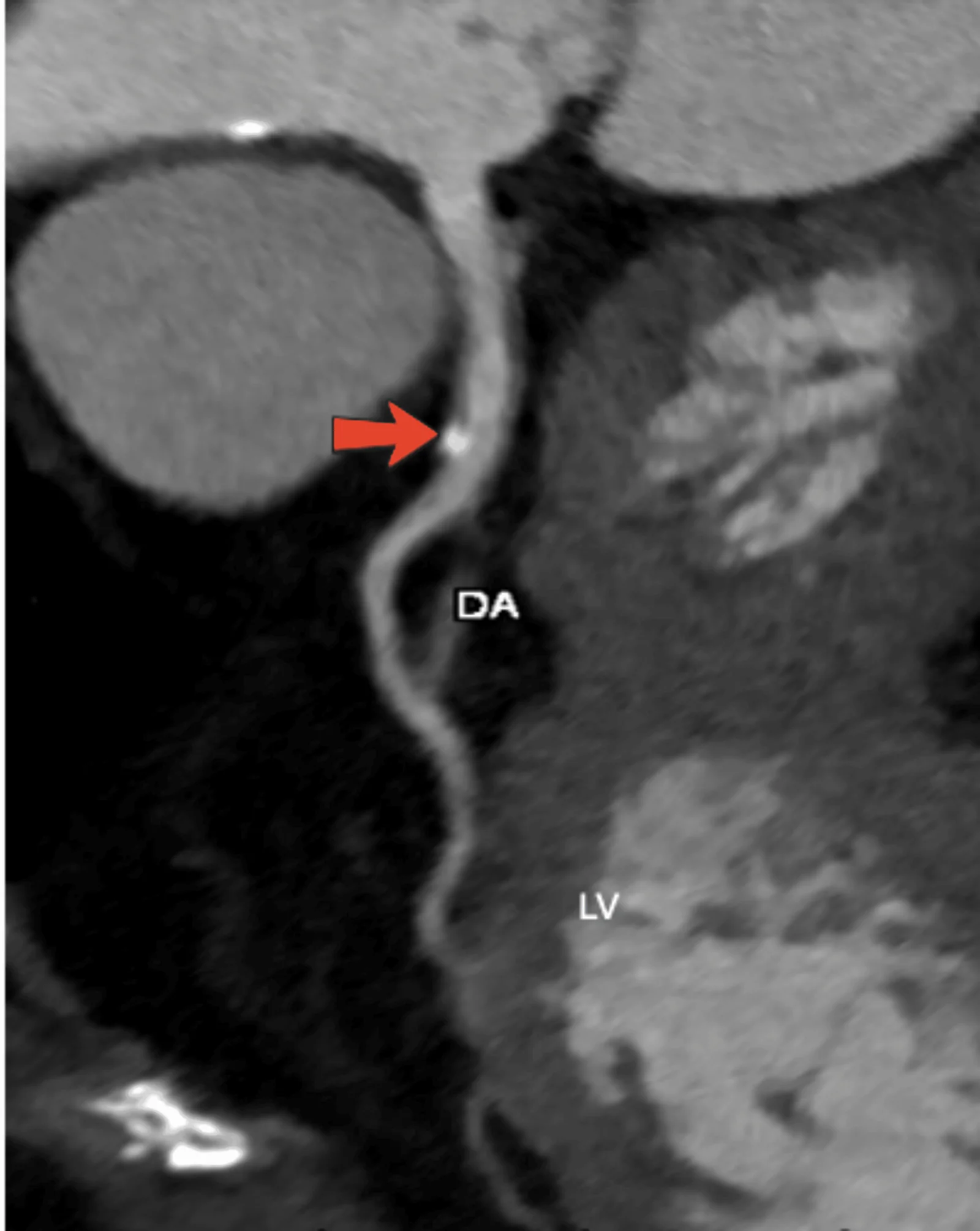
Coronary computed tomography angiography (Case 7) Coronary angiotomography showing a small plaque in the left anterior descending artery (red arrow) without perfusion impairment. LV: left ventricle; DA: left anterior descending artery

Regarding therapeutic management, beta-adrenergic bronchodilators were reduced as much as clinically feasible, and rate control was targeted using beta-blockers. One patient (Case 2) developed worsening bronchospasm following beta-blocker administration, requiring substitution with ivabradine, which achieved effective heart rate control and a favorable clinical response. One patient had known prior coronary artery disease without evidence of new acute obstructive lesions during the index episode (Case 6). Complete recovery of ventricular function was achieved in five patients, one patient demonstrated partial recovery (Case 2), and one patient died during hospitalization due to refractory ventricular fibrillation while on mechanical ventilation (Case 4).

The clinical, diagnostic, and laboratory characteristics of all seven cases are summarized in Table 1.

**Table 1:** Demographic, clinical, and laboratory characteristics of takotsubo syndrome triggered by COPD exacerbation cohort LM: left main; LAD: left anterior descending; LBBB: left bundle branch block; SVE: supraventricular ectopic beat; VE: ventricular ectopic beat; sPAP: systolic pulmonary artery pressure; N/A: not available; SABA: short-acting beta-agonist; LABA: long-acting beta-agonist; LAMA: long-acting muscarinic antagonist; EF: ejection fraction; BD: bronchodilator; F: female; M: male

Parameter	Case 1	Case 2	Case 3	Case 4	Case 5	Case 6	Case 7
Demographics							
Age (years)	74	65	81	71	75	84	69
Sex	F	F	F	F	F	M	F
Psychiatric comorbidity	Depression	Panic disorder	Depression	Depression	Early dementia	Depression	Depression
Smoking	Former	Former	Current	Former	Former	Former	Former
FEV1 (L) (% predict)	0.62L (27%)	1.29L (49%)	0.67L (34%)	0.58L (24%)	0.61L (42%)	Moderate obstruction, no BD response	0.89 (47%)
Admission							
Chest pain	No	No	Atypical	No	No	Atypical	No
Dyspnea, cough, wheezing	Yes	Yes	Yes	Yes	Yes	Yes	Yes
Heart rate (bpm)	93	129	125	140	140	84	88
Blood pressure (mmHg)	98/53	145/85	110/60	151/83	102/53	126/86	95/60
Medications							
SABA, LABA, LAMA, inhaled and systemic corticosteroid	Yes	Yes	Yes	Yes	Yes	Yes	Yes
Aminophylline / Bamifylline	Bamifylline	No	Bamifylline	Bamifylline	Bamifylline	Bamifylline	Bamifylline
Investigations							
ECG	Anterior repolarization changes	Lateral T-wave inversions	Anteroseptal ischemia	Sinus; P pulmonale; LBBB; anterior ST elevation	Sinus; frequent SVE/VE	Sinus tachycardia; frequent SVE/VE	LBBB; inverted T-waves (resolved day 7)
Troponin (ng/mL) normal < 0,16	0.51	0.16	2.05	<0.16	3.15	0.25	17.4
CK-MB (mg/L) normal < 10	7.6	20.5	12.2	22.0	11.8	5.0	5.2
BNP (pg/mL) normal < 100	113	39	523	140	1060	135	362
D-dimer normal < 500	432	644	723	N/A	300	535	1034
Prior echocardiogram	Diastolic dysfunction	Normal	None	EF 57% (dobutamine stress)	EF 57% (stress; no ischemia)	Normal	Normal
Echocardiogram at event	Apical hypocontractility; EF 48%	Septal, apical, lateral akinesia; EF 40%	Apical and anteroseptal akinesia; EF 45%	Apical and anterior/septal akinesia; EF 30%	Apical anterior, septal, lateral hypokinesia; EF 55%	Apical and anterior wall akinesia; septal hypokinesia; EF 40%; sPAP 47 mmHg	Apical akinesia; EF 45%
Follow-up echocardiogram	EF 66%; diastolic dysfunction	EF 50%; reduced akinesia area	EF 65%; no wall motion abnormalities	N/A (died)	EF 69%; diastolic dysfunction	EF 70%; no akinesia	EF 68%; normal
Coronary evaluation	No obstruction (angiography)	No obstruction (angiography)	Tortuous; no obstruction (CT angiography)	No obstruction (angiography)	No obstruction (angiography)	No acute obstruction; prior 50% LM, 40% LAD (angiography)	No stenosis; minimal LAD plaque (CT angiography)
Outcome							
Ventricular recovery	Complete	Partial	Complete	Died	Complete	Complete	Complete

Patient perspective

Patients reported increased stress and fear of cardiac death following hospitalization, with emotional repercussions extending to their family members.

## Discussion

Our findings are consistent with the description of TS reported by Templin et al. [1], including female predominance, high prevalence of psychiatric comorbidities, and the potential for serious complications, such as cardiogenic shock and mortality. However, in contrast to registry data, none of our patients reported typical chest pain when TS was precipitated by acute COPD exacerbation.

This cohort resembles the findings of Rajwani et al. [15], who described an atypical and insidious presentation of TS associated with COPD, often difficult to distinguish from isolated respiratory decompensation due to the absence of angina. Our results reinforce their conclusion that the absence of chest pain may delay recognition of TS and prompt cardiovascular management in these patients, suggesting that this presentation pattern may define a specific subset of bronchogenic TS.

The presence of COPD has been associated with a more vulnerable TS phenotype characterized by a higher risk of in-hospital complications, recurrence, and mortality, as described by Arcari et al. [2]. These data indicate that patients in our study belong to a high-risk group requiring systematic follow-up to prevent recurrence and ensure adequate rehabilitation [16,17].

In all cases, patients were exposed to intense catecholaminergic triggers, including high-dose bronchodilators and systemic corticosteroids, with one patient presenting concurrent hyperthyroidism contributing to a severe hyperadrenergic state (Case 3). These factors reinforce the pathophysiological role of sympathetic overstimulation in bronchogenic TS. The increased cardiac stress associated with beta-2 adrenergic agonist administration emphasizes the pathogenic role of sympathetic surge and highlights the need for individualized therapeutic strategies [9].

Discrepancies between modest initial BNP levels and severely depressed ejection fraction, as seen in Case 4 (BNP 140 pg/mL, EF 30%), highlight the impact of temporal discordance between biomarker sampling and imaging. Because natriuretic peptide secretion fluctuates with dynamic wall stress and gene expression, a single baseline BNP measurement may not coincide with the peak hemodynamic impairment captured on echocardiography.

Modest elevations in D-dimer levels, such as those observed in Case 3 (723 ng/mL), are common in the setting of acute COPD exacerbations due to systemic inflammation, acute phase responses, and in-hospital interventions. Rather than indicating acute pulmonary embolism, the primary clinical utility of D-dimer in this setting lies in its high negative predictive value.

Although direct catecholamine levels were not measured, a hyperadrenergic state was clinically supported by the combined exposure to potent sympathomimetic agents, including short- and long-acting beta-2 agonists (salbutamol, formoterol, and salmeterol) and methylxanthines (bamifylline), in the setting of acute respiratory stress.

Myocarditis was distinguished and excluded from TS based on the following clinical, electrocardiographic, and imaging features: 1) Serial echocardiography and wall motion recovery: All patients demonstrated a characteristic transient ballooning pattern (apical or mid-ventricular akinesia/hypokinesia with hyperdynamic basal segments) that extended beyond a single epicardial vascular territory. Crucially, repeat echocardiograms during follow-up demonstrated complete (or near-complete) regional wall motion recovery and normalization of left ventricular ejection fraction in all surviving patients, a hallmark of Takotsubo atunement rather than permanent myocarditis-induced scarring. 2) Electrocardiographic pattern: Serial ECGs showed focal or regional segmental changes (such as transient ST-segment shifts, T-wave inversions, or regional repolarization abnormalities) rather than the diffuse, non-territorial ST-T changes or PR depressions typically associated with acute myopericarditis. 3) Coronary angiography/CCTA: Invasive coronary angiography or coronary CT angiography confirmed the absence of acute obstructive coronary lesions or plaque rupture responsible for the wall motion abnormalities.

Although advanced imaging modalities such as cardiac magnetic resonance (CMR), strain echocardiography, and myocardial PET-CT can be valuable diagnostic tools when diagnostic uncertainty arises, they were not deemed strictly necessary in our cohort. The diagnosis of TS was firmly established based on the presence of typical transient regional wall motion abnormalities extending beyond a single coronary territory, the definitive exclusion of acute obstructive coronary artery disease via angiography or CCTA, and, most importantly, the documented functional recovery on follow-up echocardiography. Nonetheless, in cases where regional wall motion abnormalities or clinical presentations remain ambiguous, modalities such as CMR (for myocardial edema and characterization of late gadolinium enhancement), strain echocardiography, or myocardial PET-CT could substantially contribute to ruling out myocarditis and confirming stress-induced cardiomyopathy.

From a diagnostic perspective, the clinical overlap between acute COPD exacerbation and TS hinders early recognition. Systematic performance of electrocardiography and cardiac biomarker measurement upon admission served as a pivotal screening strategy for early diagnosis in the absence of chest pain in our cohort. Whether this approach should be adopted as routine practice in severe respiratory exacerbations warrants further prospective evaluation in larger cohorts.

To balance the conflicting demands of acute COPD bronchospasm and hyperadrenergic cardiac injury, beta-adrenergic bronchodilators were reduced to the lowest clinically effective dose. Concurrently, rate control was cautiously targeted using cardioselective beta-blockers while monitoring for airway responsiveness, substituting with ivabradine in cases of treatment-limiting bronchospasm.

Management of TS remains primarily supportive, with emphasis on reducing catecholaminergic stress. In this context, strategies include minimizing beta-agonist overexposure and judiciously introducing beta-blockers, carefully weighing risks and benefits in COPD patients [10,18]. However, in patients with COPD or asthma who develop TS, administration of beta-blockers may be strictly limited by severe bronchospasm. Ivabradine, a selective inhibitor of the sinoatrial I current, acts without affecting beta-adrenergic receptors or bronchial smooth muscle tone. Clinical evidence has confirmed its respiratory safety and absence of adverse effects on forced expiratory volume (FEV1), establishing ivabradine as a suitable alternative for heart rate control when beta-blocker therapy is contraindicated or poorly tolerated [19,20], as demonstrated in Case 2.

Non-dihydropyridine calcium channel blockers (such as diltiazem) represent an alternative rate-control option that avoids beta-2 receptor blockade in patients with reactive airway disease. However, their intrinsic negative inotropic action poses a risk of worsening hemodynamic instability in acute TS.

While beta-blocker therapy in TS patients has been associated with lower follow-up mortality, it has not consistently reduced recurrence [11,21,22]. Given that TS remains susceptible to recurrence, the identification and long-term mitigation of predisposing physical and pharmacologic factors remain essential [5,12].

Crucially, our study highlights that acute respiratory failure should not obscure concurrent cardiac pathology. The diagnostic overlap between acute COPD exacerbation and TS creates a perilous clinical blind spot, particularly because non-anginal dyspnea is frequently attributed solely to pulmonary decompensation. High clinical suspicion for concomitant acute heart failure or acute coronary syndromes must therefore be maintained during any severe exacerbation. This aligns directly with the GOLD guidelines, which explicitly acknowledge the diagnostic challenge of concurrent cardiac events during COPD exacerbations and recommend prompt evaluation with electrocardiogram, echocardiogram, cardiac biomarkers (NT-proBNP, troponin, or D-dimers), and CT pulmonary angiography to systematically exclude acute cardiac conditions or pulmonary embolism [23].

Limitations and strengths

The strengths of this study include the detailed diagnostic confirmation across all cases via invasive coronary angiography or CCTA, along with serial echocardiographic evaluation by a multidisciplinary cardiology and pulmonology team. Limitations include the retrospective single-center design and limited sample size, which preclude multivariable risk modeling. Additional limitations include the lack of systematic data on arterial blood gases, static lung volumes, and diffusion capacity (DLCO). Although the ECG and imaging features strongly favor TS and render infectious myocarditis unlikely, we acknowledge as a study limitation that CMR imaging, which is recommended by expert consensus for definitive myocardial tissue characterization, was not performed in our cohort. Therefore, while clinical and conventional imaging findings strongly support the diagnosis, the absence of CMR precludes the absolute, definitive exclusion of myocarditis. Prospective multi-center registries are required to further elucidate optimal management protocols for TS secondary to respiratory failure.

## Conclusions

TS can be precipitated by acute COPD exacerbations, presenting a diagnostic challenge when respiratory symptoms predominate in the absence of chest pain. While systematic electrocardiographic and cardiac biomarker evaluation may aid early recognition in selected patients, our findings from this seven-patient observational case series cannot establish the definitive effectiveness of routine cardiac screening protocols or specific therapeutic interventions. Further prospective, multi-center studies are warranted to validate these approaches in this vulnerable population.
